# The osteogenic differentiation of human dental pulp stem cells in alginate-gelatin/Nano-hydroxyapatite microcapsules

**DOI:** 10.1186/s12896-020-00666-3

**Published:** 2021-01-11

**Authors:** Mahdieh Alipour, Nima Firouzi, Zahra Aghazadeh, Mohammad Samiei, Soheila Montazersaheb, Ali Baradar Khoshfetrat, Marziyeh Aghazadeh

**Affiliations:** 1 Dental and Periodontal Research Center, Faculty of Dentistry, Tabriz University of Medical Sciences, Tabriz, Iran; 2 Stem Cell and Tissue Engineering Research Laboratory, Sahand University of Technology, Tabriz, Iran; 3 Stem Cell Research Center and Department of Oral Medicine, Faculty of Dentistry, Tabriz University of Medical Sciences, Tabriz, Iran; 4 Department of Endodontics, Faculty of Dentistry, Tabriz University of Medical Sciences, Tabriz, Iran; 5 Molecular Medicine Research Center, Tabriz University of Medical Sciences, Tabriz, Iran

**Keywords:** Alginate-gelatin microcapsules, Bone tissue engineering (BTE), Human dental pulp stem cells (hDPSCs), Nano-hydroxyapatite

## Abstract

**Background:**

Microcapsule is considered as a promising 3D microenvironment for Bone Tissue Engineering (BTE) applications. Microencapsulation of cells in an appropriate scaffold not only protected the cells against excess stress but also promoted cell proliferation and differentiation. Through the current study, we aimed to microcapsulate the human Dental Pulp Stem Cells (hDPSCs) and evaluated the proliferation and osteogenic differentiation of those cells by using MTT assay, qRT-PCR, Alkaline phosphatase, and Alizarine Red S.

**Results:**

The SEM results revealed that Alg/Gel microcapsules containing nHA showed a rough and more compact surface morphology in comparison with the Alg/Gel microcapsules. Moreover, the microencapsulation by Alg/Gel/nHA could improve cell proliferation and induce osteogenic differentiation. The cells cultured in the Alg/Gel and Alg/Gel/nHA microcapsules showed 1.4-fold and 1.7-fold activity of BMP-2 gene expression more in comparison with the control group after 21 days. The mentioned amounts for the BMP-2 gene were 2.5-fold and 4-fold more expression for the Alg/Gel and Alg/Gel/nHA microcapsules after 28 days. The nHA, addition to hDPSCs-laden Alg/Gel microcapsule, could up-regulate the bone-related gene expressions of osteocalcin, osteonectin, and RUNX-2 during the 21 and 28 days through the culturing period, too. Calcium deposition and ALP activities of the cells were observed in accordance with the proliferation results as well as the gene expression analysis.

**Conclusion:**

The present study demonstrated that microencapsulation of the hDPSCs inside the Alg/Gel/nHA hydrogel could be a potential approach for regenerative dentistry in the near future.

**Graphical abstract:**

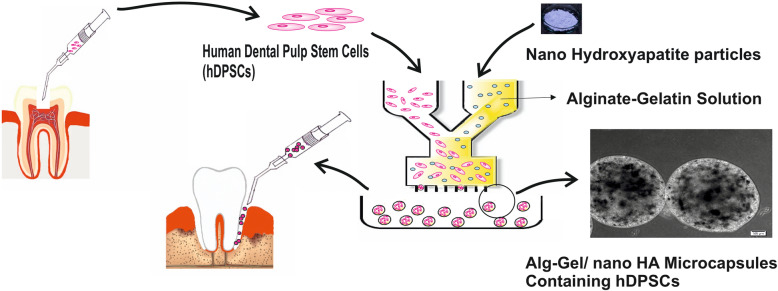

## Background

Congenital and acquired bone defects and diseases led to the significant morbidity and reduction of quality of life for a wide range of patients. Bone defects in the critical sizes remained challenging for the dentist and orthopedic surgeons [1]. Allogenic and xenogeneic bone grafts were available sources, but infection transmission and limited efficacy were the main disadvantages [2]. The autologous bone grafts were commonly used for the defects and considered as a gold standard method [3]. However, the limitations of the grafts such as donor site morbidity as well as increased operative time restricted the method application [4].. Designing the natural and synthetic scaffolds with the capability to deliver the growth factors for stem cell growth and differentiation is a novel strategy that can overcome those drawbacks in the Bone Tissue Engineering (BTE) [5, 6]. Although cell-based therapies play an important role in regenerative medicine, there is a major concern for the efficient delivery and resistance of the transferred cells after injection or implantation. One of the novel advanced methods as a solution can be tissue modification with the fabrication of the modular block [7].

The appropriate scaffold as a key part of tissue engineering should provide cell survival, induce cell bioactivity, and enhance cell retention in the implanted sites. Encapsulation of the cells with various polymers protects them from injection forces and immune system reactions while allowing the bidirectional diffusion of the nutrients and wastes [8–10]. Microcapsules, as the spherical micro carriers, have been recently used for providing a three-dimensional hydrophilic microenvironment for supporting and transferring the stem cells to the site of defects [11–13]. The current technique has been shown successful in vitro and in vivo results for the treatment of diabetes, liver dysfunction, and neurological disease [14–18].

Bone has a highly complex hierarchical structure containing a porous composite of hydroxyapatite and collagen [19]. Bone Marrow Mesenchymal Stem Cells (BMSCs) are the major sources for bone tissue engineering. However, the morbidity of the donor site and the painful harvesting methods of these cells have limited the application of the cells [20, 21].

Therefore, Human Dental Pulp Stem Cells (hDPSCs) were introduced as the multipotent stem cells with self-renewal ability by Gronthos et al. The mentioned cells could be harvested from both primary and permanent teeth during the routine teeth extraction for the orthodontic treatment [22]. For bone tissue engineering, DPSCs showed more proliferation ability and number of colony clusters compared to BMSCs [23]. Although other sources of mesenchymal stem cells are applied for the regeneration of hard tissues, the complications of their harvesting methods have limited their applications in practice. As mentioned DPSCs are an easy-access source of mesenchymal stem cells that has similar characteristics to bone marrow stem cells for hard tissue regeneration. Due to the availability and notable differentiation potential, the application of these cells for tissue engineering has attracted enormous attention in recent years. These cells had considerable results in in vivo studies for the treatment of muscular dystrophy, corneal injuries, limb ischemia, Alzheimer’s, and Parkinson’s disease. These cells were studied several times for bone tissue engineering in animal models and revealed positive results. According to the great differentiation ability of these cells, it is important to carry them safely to the target tissues and provide a sustained but effective environment for their differentiation [23–30].

Alginate polysaccharide is a member of the linear polymeric acid groups, which were isolated from the brown sea algae [3, 31]. Alginate is a biopolymer contains G monomers (G block) which include a high affinity to Ca^2+^ [32]. Alginate properties such as degradability in the physiologic situation, low toxicity, and ability to support the deposition of a calcified matrix, turn it into an important material in bone tissue engineering [33, 34]. Also, the alginate matrix is a porous and hydrophilic structure, which allows oxygen, nutrients, and wastes transportation [32]. The ability of the alginate to make hydrogels in the presence of the cations as calcium and barium makes it one of the polymers of choice in cell and protein delivery, tissue engineering, and wound dressing material [35]. The stated material has been widely used as the main component for the capsulation of the different cells [36–38].

Gelatin is a natural polymer with a similar composition to collagen, which could provide the proper structure for BTE. The development of the suitable conditions in the deposition and nucleation mineral phase, in addition to the availability and low costs compared with the collagen, makes gelatin a component of choice in bone tissue engineering [39–41].

Bioactive materials used in BTE should enhance the osteoinductivity of the scaffold [42, 43]. Nano-hydroxyapatite (nHA) is the main inorganic component of bone matrix embedded in the organic component of natural bone (collagen type I) [44, 45]. Due to outstanding features such as biocompatibility, osteoconductivity, non-toxicity, and the ability to be resorbed into bone tissue, nano-hydroxyapatite could improve bone regeneration when incorporated with scaffolds. Nano-hydroxyapatite crystals increase the strength of collagen fibers, facilitate and promote bone formation with increasing cell adhesion. Also, hydroxyapatite improves the deposition of mineral compositions contain calcium ions [46–48]. Studies showed that nano-hydroxyapatite particles increased surface roughness, which improved the absorption of chemical species from the surrounding environment [49, 50].

In our previous studies, the osteogenic potential of the Alginate/Gelatin/nano-hydroxyapatite (Alg/Gel/nHA) microcapsule for the modular bone formation was evaluated by using the osteoblastic cell line [6, 44]. Through the present investigation, the Alginate/Gelatin (Alg/Gel) and Alginate/Gelatin/nano-hydroxyapatite (Alg/Gel/nHA) microcapsules containing hDPSCs were used to investigating the influence of 3D spherical scaffold and nano-hydroxyapatite (nHA) on the cell proliferation and osteogenic differentiation of the stem cells for bone regeneration. In this regard, hDPSCs were capsulated in the Alg/Gel/nHA for the first time in the current research. In order to the enormous expansion in the regenerative dentistry, especially bone regeneration in the maxillofacial region, the easy-accessible and available stem cells in the mentioned region could be a promising source. Moreover, the application of the injectable scaffolds for transferring of hDPSCs could protect the cells and provide an appropriate environment for the induction of mineralization in the cells. The present study would illuminate the hDPSCs phenotype in 3D microbeads and its potential toward the formation of the modular bone tissues for future in vivo studies.

## Results

### Influence of the hydrogel composition on the microcapsule stability and proliferation of microcapsulated hDPSCs 

**Fig. 1:**
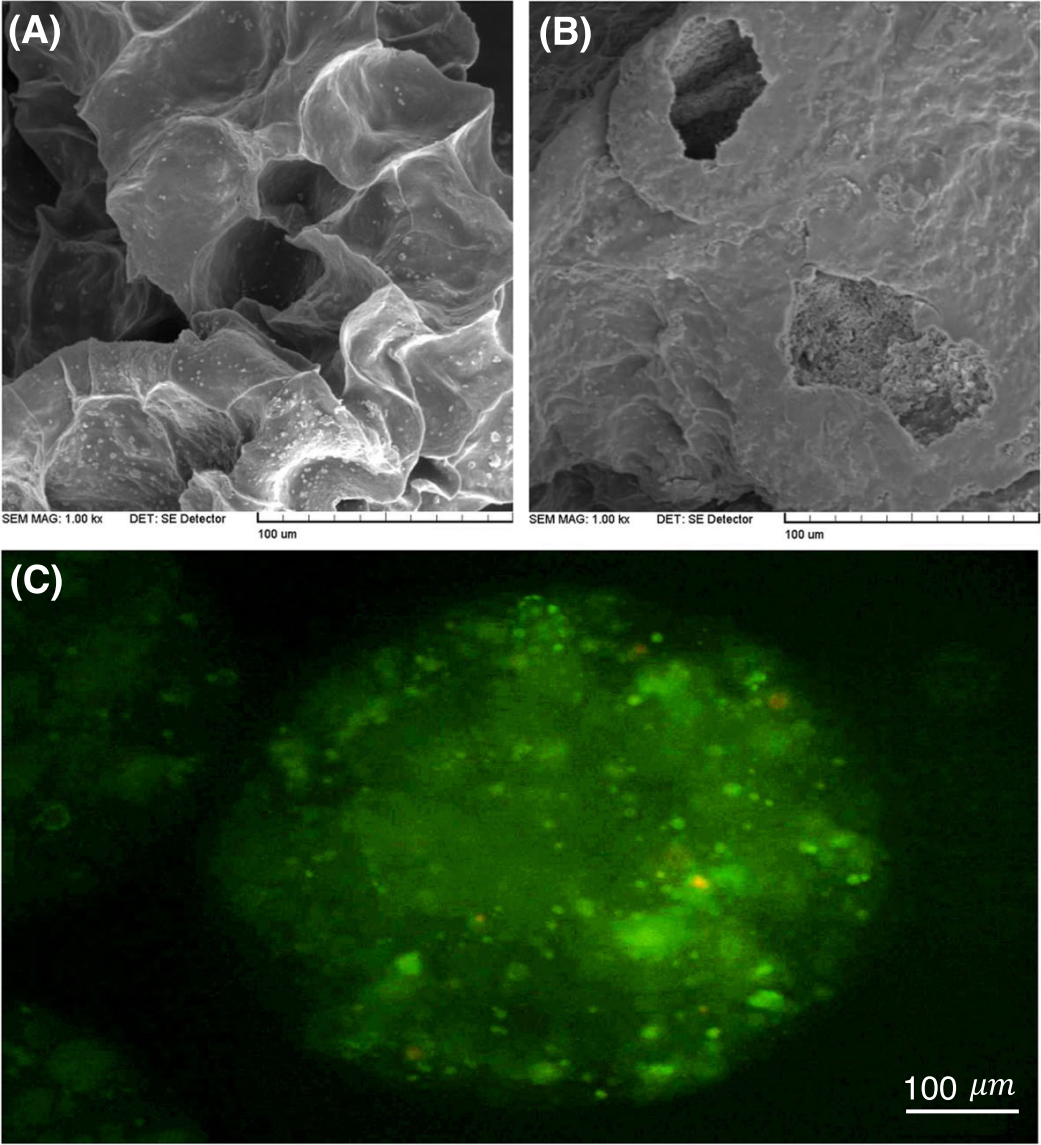
SEM images of microcapsule surface after gelation for hydrogel compositions of Alg/Gel (**a**), Alg/Gel/nHA (**b**) and live/dead staining for the cells microencapsulated at Alg/Gel/nHA after 21 days (**c**) (Scale bar: 100 *μ* m)

Figure 1 reveals the surface morphology of both hydrogel microcapsules of Alg/Gel (Fig. 1a) and Alg/Gel/nHA (Fig. 1b) as well as the appearance of the proliferated hDPSCs in the microcapsules (Fig. 1c). Interestingly, the Alg/Gel microcapsules containing nHA showed a rough and more compact surface morphology in comparison with the Alg/Gel microcapsules (Fig. 1 a-b) revealing microcapsule stability can be improved when nHA is added to the alginate-based hydrogels.

**Fig. 2:**
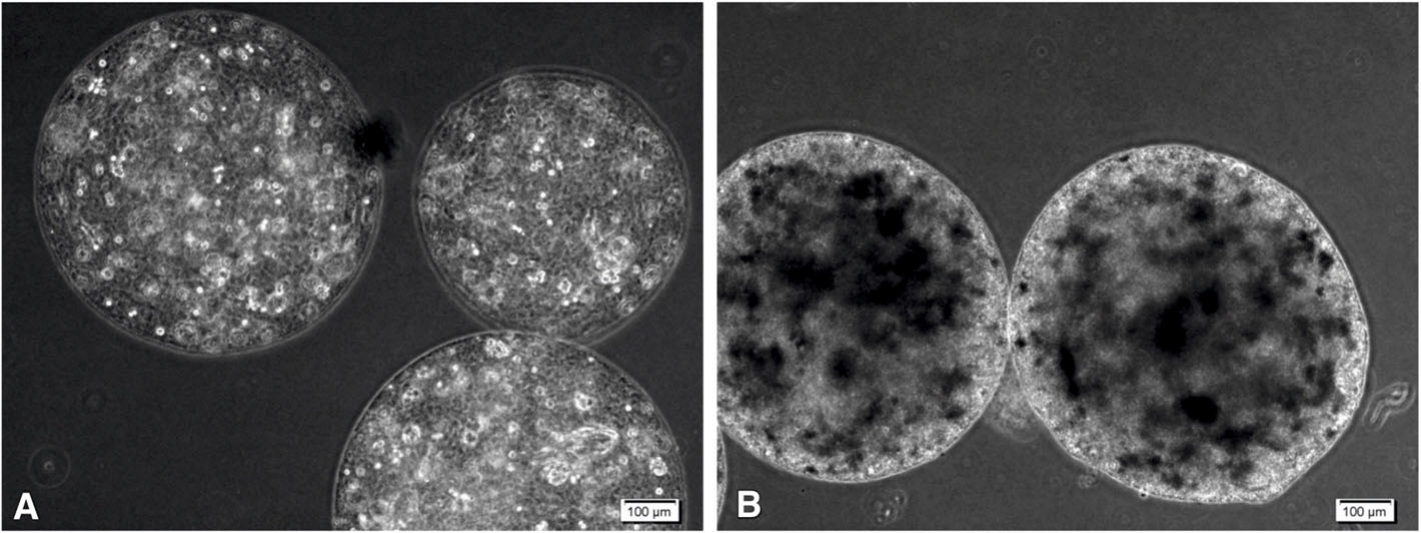
Microscopy images of microcapsules containing hDPSCs after 21 days. (**a**) Alg/Gel microcapsules containing hDPSCs (**b**) Alg/Gel/nHA microcapsules containing hDPSCs

Live and dead assay (Fig. 1c) showed cell proliferation in the majority of the fabricated microcapsules. However, some of the cells died inside the bigger aggregates of the microcapsules. The cell viability in the micro-carrier structures can be affected by the sizes of more than 400 *μ* m . The highly hydrated 3-D alginate-based microbeads, as shown in Fig. 1c, could provide an immobilized matrix for the cells with a permeable membrane for waste, nutrients, and oxygen transmissions. Therefore, the average diameter of the microcapsules was examined by BEL view software (ver.6.2) (data are not shown). More than 65% of the microcapsules in both cases had 300±40 μm diameter leading to sufficient nutrient and waste transmissions to the inner core cells. The microscopy images of the microcapsules were shown in Fig. 2.

**Fig. 3:**
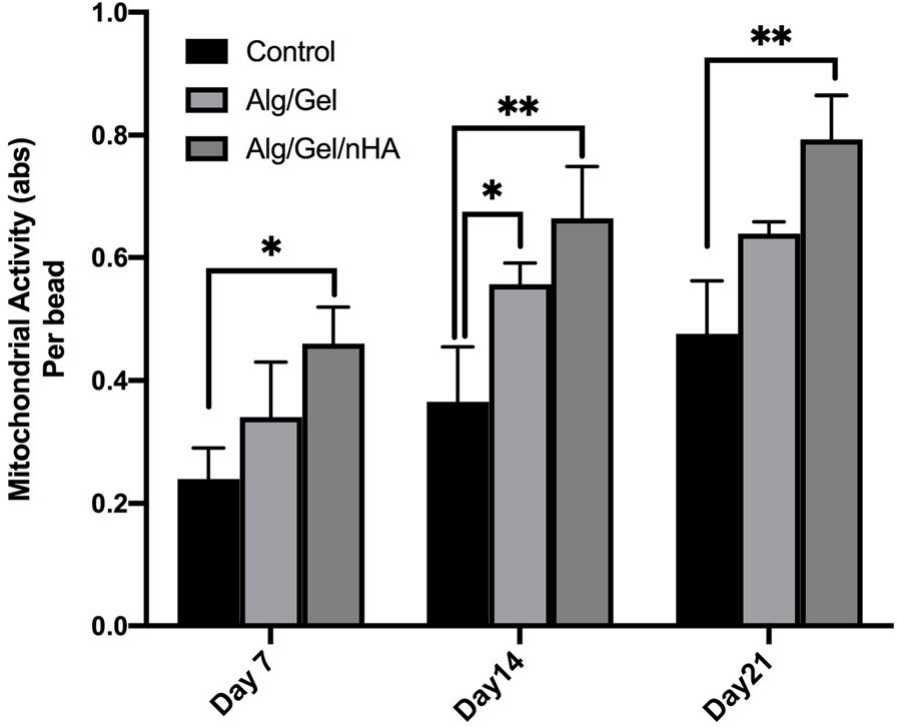
MTT assay results for Alg/Gel and Alg/Gel/nHA microcapsules after 7, 14, and 21 days (*P< 0.05) and (**P< 0.01)

Mitochondrial activity as a vital cue demonstrates the viability of cells. Human dental pulp stem cells were cultured in both nHA modified and unmodified Alg/Gel microcapsules (Fig. 3). Unmicrocapsuled stem cells cultured on the T-flasks were considered as a control group. Figure 3 shows the proliferation of the hDPSCs in the microcapsules and without microcapsules for a 3-week culture period. The proliferation of the stem cells increased in the microcapsules and on the control group during the considered period. Dental pulp stem cells in the microcapsules revealed statistically more mitochondrial activities as compared to the control group during the culture period (*P* < 0.05). Interestingly, the microcapsules containing nano-hydroxyapatite showed significant increases in all experiment times. Using nano-hydroxyapatite in the microbeads increased the mitochondrial activity of the stem cells 1.26 times (*P* < 0.05) per microcapsules after 3 weeks. The obtained outcomes can be accomplished that nano-hydroxyapatite plays an important role in the proliferation of dental pulp stem cells.

### Influence of the microcapsule composition on bone-related gene expressions of the microencapsulated hDPSCs

The qRT-PCR analysis was performed to evaluate the effect of the Alg/Gel and Alg/Gel/nHA microbeads on the expression levels of the key genes in bone and dentin formation including osteocalcin, BMP2, RunX2, osteonectin, and DSPP after 21 and 28 days.

**Fig. 4:**
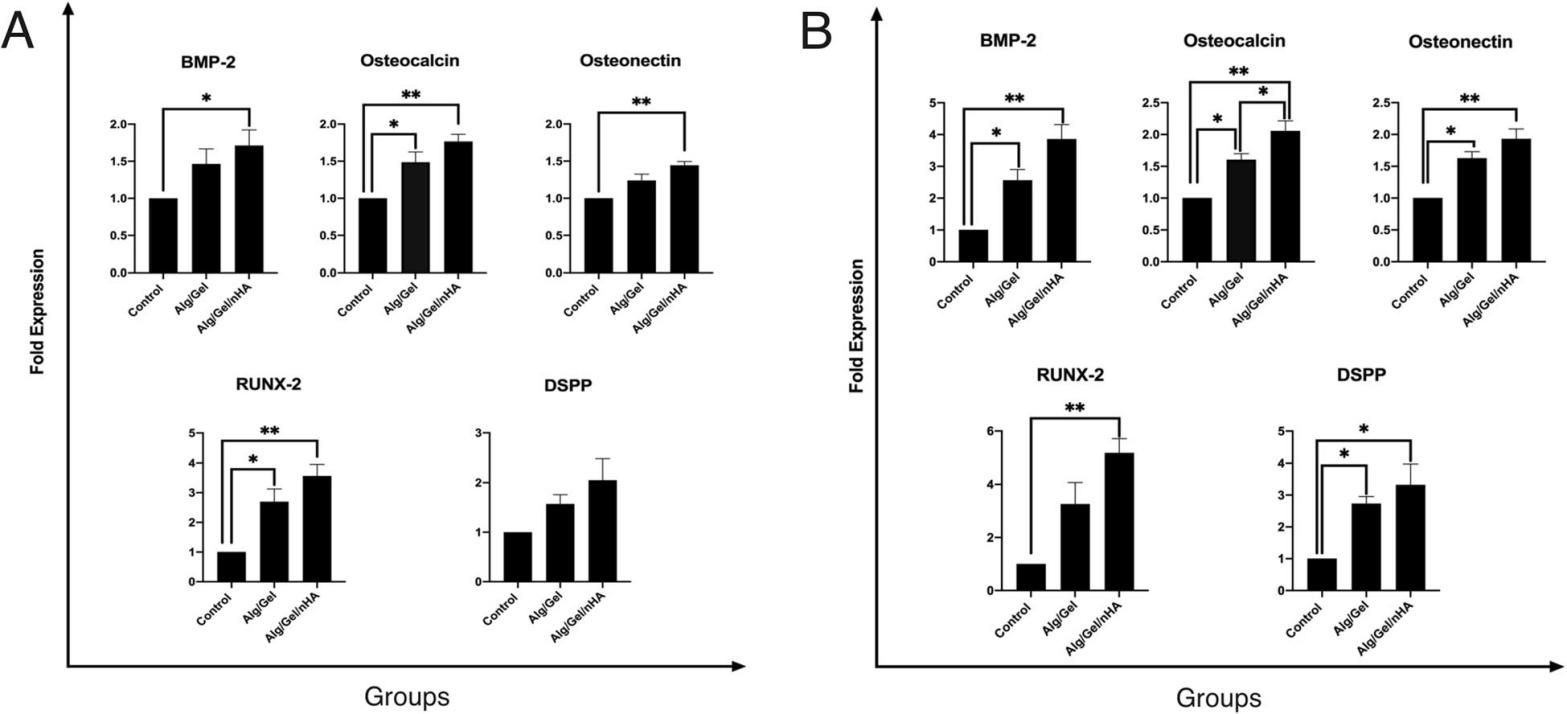
The expression levels of osteogenic differentiation gens of hDPSCs grown on T-flask, Alg-Gel, and Alg-Gel-nHA microcapsules (**a**) after 21 days and (**b**) after 28 days. (*P< 0.05) and (**P< 0.01)

According to the results shown in Fig. 4, the expression levels of all the genes increased in the hDPSCs, which were microcapsulated in the Alg/Gel and Alg/Gel/nHA as compared to the control group.

on day 21, BMP-2 levels significantly increased only in the Alg/Gel/nHA group. However, on day 28, the expression levels were significantly higher in both groups comparing the control group. By comparing the BMP-2 expression in two distinct days, it could figure out that the expressions of this osteogenic gene in microbeads, especially in the presence of nano-hydroxyapatite were up-regulated.

After 21 days microcapsulation, osteocalcin expression in both microcapsule groups was significantly higher than the control group. Although the expression levels in the Alg/Gel/nHA hydrogel microcapsule were higher than the Alg/Gel groups after 21 and 28 days, the mentioned difference was statistically significant only on the 28th day (*P* < 0.05).

After 21 days, osteonectin expression in the Alg/Gel microcapsules was in the range of the control sample, while a significant increase of the gene expression was observed in the Alg/Gel/nHA group. However, after 28 days, both microcapsule groups showed significant increases in the osteonectin expression levels in comparison with the stem cells without microcapsules.

In a similar manner, it can be imagined that the concentrations of both osteocalcin and osteonectin can be intensified by the passing of time. These results provided confidence that the cell-laden Alg/Gel/nHA microcapsule can create bone volume in in vivo experiments.

RUNX-2 expression of the hDPSCs in the Alg/Gel/nHA microcapsules showed a 3.5-fold increase as compared to the control group on the 21st day, the value of which was about 2.6-fold for the Alg-Gel microcapsule. However, after 28 days, the significant RUNX-2 up-regulation was observed in the Alg/Gel/nHA group which was 5.1-fold more than the control group (*P* < 0.01).

The results revealed that a three-dimensional microenvironment created by alginate-based microcapsules containing nHA could up-regulate RUNX-2 expression considerably in comparison with the conventional two-dimensional control group.

As shown in Fig. 4, the DSPP expression in both the Alg/Gel/nHA and Alg/Gel microcapsule groups showed no significant differences between the groups after 21 days. The expression of the DSPP gene on the 28th day for both the microcapsule groups, however, was significantly increased, the value of which was 2.7-fold and 3.3-fold higher than the control group. The low up-regulation of this gene as a classic marker of the odontogenic differentiation compared to other osteogenic genes demonstrated the useful application of the microcapsulation of the hDPSCs with Alg/Gel/nHA in bone tissue engineering.

### Influence of the microcapsule composition on the osteogenic marker production and mineralization of hDPSCs

**Fig. 5:**
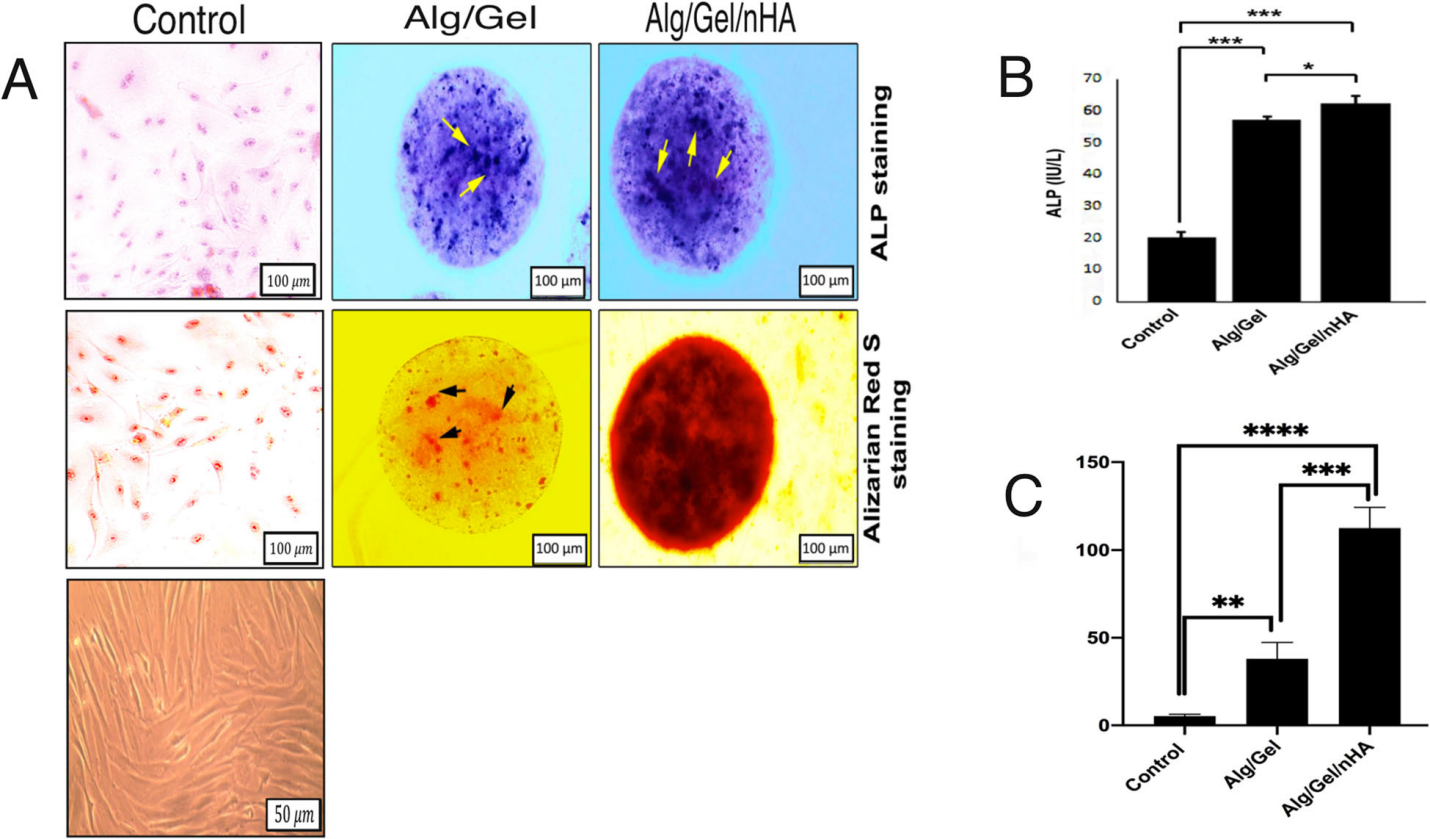
**a** Calcium deposition and ALP excretion of the hDPSCs cells in the Alg/Gel microcapsules and Alg/Gel/nHA microcapsules. **b** ALP Enzyme Activity of the hDPSCs grown on the culture medium (control), Alg/Gel microcapsules, and Alg/Gel/nHA microcapsules after 21 days. **c** Quantitative analysis of the alizarin red staining was performed using ImageJ software. *P< 0.05, **P< 0.01, *** *P*< 0.001, and **** *P*< 0.0001

To validate the presence of the osteocalcin and bone sialoprotein, two distinct stainings were carried out. The enzymatic activity could be asserted directly for the dyed cells. As shown in Fig. 5a, blue-color spots indicated the positive ALP activity in the fixed days. Intensified hued microbeads with nano-hydroxyapatite presence significantly aggravated hydrolase enzyme expression, motivating ALP expression at the surface of the hDPSCs, as compared to the Alg/Gel microbeads.

Quantification of alkaline phosphatase measured in the microcapsules revealed further corroborating evidence for the hDPSCs differentiation. According to the ALP activity shown in Fig. 5b, differentiation of the hDPSCs to osteoblast-like cells increased significantly 2.7-fold and 3-fold in the Alg/Gel and Alg/Gel/nHA microcapsules, respectively. The increases in the ALP levels as early bone formation indicator in the Alg/Gel/nHA were statistically higher as compared to the Alg/Gel microbeads (*P* < 0.05). In fact, the microspheres create the three-dimensional space for cells to be in the exposure of the factors that accelerate the differentiation as well as retain cells phenotype. The quantitative analysis confirmed that the ALP enzyme activity increased remarkably in the alginate-based microcapsules in comparison to the non-3D scaffolds.

As also illustrated in Fig. 5a for calcium deposition, arrows show the red-colored surroundings nodules, which demonstrated the mineralized cells in the microcapsules. Hydroxyapatite particles in the presence of Alg/Gel composition increased the calcium deposition on days 21.

The results also confirmed the formation of the mineralized matrix and osteoblastic differentiation in both the microcapsules.

Consequently, it can be deduced that the 3-D microsphere network provided a network for cells to precipitate the calcium and relevant enzymes content over time to speed up the modular formation.

The statistical analysis showed the significant increases in the red color intensity of the Alg/Gel/nHA microcapsules compared to the other groups, which revealed the stronger mineralization in that group.

## Discussion

Microcapsules are widely used in tissue engineering including regeneration of the different tissues and organs such as liver, cartilage, skin, neural tissue, and bone [9, 51–55]. These microstructures with different characteristics including immunoisolation, micrometer size, and providing 3D microenvironment were considered as a promising approach in regenerative medicine [9, 56]. The modular micro carriers could directly be injected and transplanted to the defect side which is important for the reconstruction of the hard tissues [57]. That property is important for the reconstruction of the hard tissues. The injectable micro carriers could be adjusted to the bone defects with the irregular shape and geometry, occupying the available spaces, precluding the invagination of the adjacent tissues, and promoting the tissue repairing [58, 59]. As shown in the graphical abstract, the current study aimed to use the available source of the stem cells in the oral cavity and microcapsulation of these cells for the modular tissue engineering approach of the bone defects in the oral and maxillofacial region.

The main findings of the current study are the high osteogenic differentiation capacities of the hDPSCs in Alg/Gel/nHA microcapsules. Moreover, both ALP and Alizarin red staining of the micro carriers showed a greater extent of the mineral deposition in nano-hydroxyapatite modified microcapsules.

Through the current study, the researchers investigated the osteogenic potential of the hDPSCs as an available source of the mesenchymal stem cells from the orofacial region in the injectable Alg/Gel/nHA microcapsules for the first time, which may be used for regeneration of hard tissues such as bone in the same region.

Alginate is a natural biopolymer that has been widely used for drug delivery, dental impression materials, and tissue engineering [4, 60, 61].

Gelatin is derived from collagen, the major organic component of the extracellular matrix of bone tissue, with adequate properties for bone tissue regeneration and revascularization [62, 63].

In the structure of Alg/Gel microcapsules, a rapid reaction between carboxyl groups of alginate and barium cations makes it possible to form an ion-crosslinking hydrogel. Microcapsules were cross-linked by the ionotropic gelation in the presence of BaCl_2_ that has shown high stability and low degradability in comparison to CaCl_2_ [64]. Indeed, adding nHA to the system increases the crosslinking of the polymeric network, leading to an enhanced homogeneity and strength of the microcapsules [58].

Nano-hydroxyapatite (nHA) as the major inorganic mineral part of bone could increase the homogeneity and strength of microcapsules. Moreover, nHA increased the crosslinking of the polymeric network [58]. These features besides, the facilitation of bone formation in the presence of nHA turn it into an important component of BTE [32].

The interaction between barium ions of nHA and G-blocks of alginate makes a strong matrix with the rougher surface which improves cell adhesion, proliferation, and differentiation compared to a smooth alginate surface. In the current study, the proliferation of hDPSCs in the Alg/Gel/nHA microcapsules was higher than Alg/Gel and control groups. Moreover, the cell viability in the micro-carrier structures was influenced by the size and mass too, which can diffuse inner layers [65]. The viability and proliferation of hDPSCs in Alg/Gel and Alg/Gel/nHA microcapsules increased during the study. This viability is a result of an appropriate bead size, which could provide an immobilized matrix with a permeable membrane that facilitates waste, nutrients, and oxygen transmission for cells. This could avoid the crowd of toxic waste and lead to appropriate activity and proliferation of cells. These results were in accordance with other previous studies that used microcapsulation methods for cell transferring [7, 32].

The aim of BTE is not only to provide 3D structures and cell proliferation without cell cytotoxicity or foreign body reaction but also the regeneration of new bone by provoking osteogenic differentiation of stem cells [4, 66].

In the oral and maxillofacial regions for the regeneration of bone defects, the non-invasive injectable methods for stem cell delivery are more useful [67]. The osteo/odontogenic differentiation of hDPSCs on various scaffolds was evaluated previously [47, 49, 68, 69]. However, none of these scaffolds evaluated non-invasive injectable carriers for these cells. In the current study, we transferred these easy-accessible stem cells in the oral region to spherical injectable microcapsules, which could be useful for bone regeneration of this area.

The osteogenic differentiation of the hDPSCs in Alg/Gel and Alg/Gel/nHA group was assessed by the relative expression of the BMP-2, Osteocalcin, Osteonectin, and RUNX-2. Various extracellular ligands such as BMPs, WNTs, and FGFs control the osteogenic differentiation of the different multipotent mesenchymal stem cells [70]. These ligands direct the three main stages of the osteogenic differentiation, which is associated with the expression of some genes like BMP-2, RunX-2, Osteocalcin, and Osteonectin [71].

The expression of the BMP-2 as the most important growth factor in bone formation is known as an early indicator of the calcified tissue generation and osteoblastic differentiation [66, 72]. The expression of the factor was significantly higher in Alg/Gel/nHA group after 21 and 28 days.

Osteocalcin and osteonectin are noncollagenous proteins in the extracellular matrix of bone. Osteocalcin has an important role on the maturation of the mineralized tissues and the regulation of the osteogenic differentiation of the mesenchymal stem cells [73]. The microcapsulation of the hDPSCs has positive effects on the expression levels of the genes. However, these effects were greater in the Alg/Gel/nHA group.

RUNX-2 is known as a critical transcription factor associated with bone formation and plays an important role in the differentiation of the pre-osteoblastic cells to mature osteoblasts. Also, this factor upregulates the VEGF factor which is important in the angiogenesis of the bone tissue [35, 74]. The upregulation of this gene as a late indicator of osteogenesis was higher than the other genes after 21 and 28 days.

Moreover, DSPP gene expression, a classic odontogenic differentiation marker, was assessed in order to investigate any odontogenic differentiation of the hDPSCs in microcapsules. As demonstrated in the results, the up-regulation of this gene did not increase as well as the osteogenic genes in the microcapsules, which indicated the osteogenic potential of the Alg/Gel/nHA microcapsules rather than the odontogenic potential.

In general, the hDPSCs in the Alg/Gel/nHA microcapsules showed a higher expression of the osteogenic genes after 21 and 28 days. The obtained results complied with the other previous studies, which evaluated the different alginate- nano hydroxyapatite-based scaffolds on the osteogenic differentiation of the stem cells [58, 66, 75].

The combination of the calcium with alizarin red makes the orange nodules, which showed the mineralization sites [47]. These nodules were clearly observed in the Alg/Gel/nHA group.

ALP enzyme activity as a major factor in the beginning phase of bone matrix mineralization was quantified by the hydrolysis of p-nitrophenyl phosphate to p-nitrophenol [76]. This enzyme is a well-known marker for the indication of mineralization. ALP deposition occurs during osteoblast maturation and bone matrix mineralization [77]. Therefore, the ALP activity could demonstrate the biological activity of osteoblasts [47]. Moreover, The formation of the bone-like mineralized tissue due to calcium deposition is essential for bonding of newly regenerated bone with the former bone tissue [58, 78].

The results observed for the physical and osteoconductive properties of the nHA particles in the hDPSCs-laden alginate hydrogels were in agreement with the reported results of the previous studies [6, 26, 41, 44], indicating the importance of the nHA bioactivity in the composite hydrogel.

To evaluate the mineralized deposition of the hDPSCs in Alg/Gel/nHA microcapsules, alkaline phosphatase and alizarin red staining were performed in the current study. The formation of the mineralized nodules as shown in our results indicated the late stage of the osteogenic differentiation. In summary, our results indicated the notable enhancement in mineralization of the hDPSCs in Alg/Gel/nHA microcapsules after 21.

In general, our results showed that the proliferation of the hDPSCs increased and the expression of all osteogenesis genes were upregulated in the nano-hydroxyapatite containing microcapsules. Moreover, calcium deposition increased in the presence of nHA. The current study evaluated the Alg/Gel/nHA hydrogel microcapsules microcapsules in the osteogenic differentiation of the hDPSCs for the first time, based on the our best knowledge.

## Conclusions

The cell viability and proliferation of the hDPSCS increased in both Alg/Gel and Alg/Gel/nHA microcapsules. Besides, the differentiation of the hDPSCs was immoderately intensified in the presence of the nano-hydroxyapatite. In the engineering of the hard mineralized tissues, the structure of the carriers must be hardened in order to simulate the real extracellular matrix. The presence of the nano-hydroxyapatite particles in microcapsules simulated the real architecture of the bone tissue. In addition, these engineered scaffolds allowed the hDPSCs to express the osteogenic markers more than the control group. The present study demonstrated that Alg/Gel/nHA hydrogel microcapsule containing easy-accessed stem cell sources in the oral cavity as a potential bone building block for the regeneration of the bone loss and defects in the orofacial region.

## Methods

### Materials

Gelatin (type A, from porcine skin, 300 bloom), nHA (average size 100 nm), alginic acid sodium salt (from brown algae, medium viscosity), Alizarin red S, propidium iodide (PI), BCIP/NBT (5-Bromo-4-chloro-3-indolyl phosphate/Nitroblue tetrazolium) and barium chloride were purchased from Sigma-Aldrich. Trypsin, fetal bovine serum (FBS), high glucose Dulbecco’s modified Eagle’s medium (DMEM/HG), and penicillin/streptomycin were obtained from Gibco (Singapore). 3-(4, 5-Dimethylthiazol-2-yl-2, 5-diphenyltetrazolium bromide) (MTT) and Trizol reagent were supplied from Invitrogen (Carlsbad, CA, USA). Complementary DNA (cDNA) synthesis kit and SYBR Green PCR Master Mix were purchased from Yekta Tajhiz Company (Tehran, Iran).

### Isolation and adhesion of human dental pulp stem cells (hDPSCs)

This study was approved by the stem cell research center of Tabriz University of Medical Sciences under the code of TBZMED.REC.1396.654. All experimental protocols were in compliance with the Helsinki declaration.

Human dental pulp stem cells were isolated from extracted permanent teeth according to orthodontic treatment in the Oral and Maxillofacial Surgery Department of Dental Faculty of Tabriz University of Medical Sciences. All participants signed the written consent after being informed about the objective of the study.

Isolation and characterization of the hDPSCs were performed as our previous study [79]. Briefly, after teeth extraction under local anesthesia, teeth were split with a chisel and the extracted pulp tissue was divided into small pieces. Pulp pieces were then digested in 3 mg/mL type I collagenase and 4 mg/ml dispase for 40 min at 37 °C. After centrifuging, the harvested cells were cultured to reach 80% confluence. At the third passage, the cell suspension was transferred to round end bottom tubes and stained with immunoglobulin G–fluorescein isothiocyanate-conjugated or phycoerythrin-conjugated anti- CD73, CD90, CD105, CD166, CD34, CD11b, and CD45 (Beckman Coulter, Villepinte, France, 20 mL each). After that, the cells were washed by fluorescence-activated cell sorting (FACS) wash solution and centrifuged for 5 min.

Dental pulp stem cells were transferred to 75 cc T-flasks coated by polystyrene surface containing DMEM high glucose amplified with 10% fetal bovine serum (FBS), 100 U/mL penicillin/ streptomycin, and 1% amphotericin B for proliferation.

### Microcapsule fabrication and analysis

Powder of the gelatin and nano-hydroxyapatite were sterilized by autoclaving and alginic acid sodium salt was suspended in ethanol 70% and kept under the laminar hood for 24 h.

Cell microcapsulation was carried out according to our previous study [7]. Concisely, 2% w/v sodium alginate with and without 2% w/v nHA and sterilized gelatin were dissolved in the calcium-free Krebs Ringer HEPES-buffered saline (CF-KRH, pH = 7.4). Microcapsules preparation was carried out by the voltage power supply and syringe pump (Vita Teb, Iran). 2× 10^6^ cells/ml were mixed with alginate (1%) solutions containing gelatin (1.25%) with or without nHA (1%) and loaded into the syringes equipped with 30-gauge needles. Extruded microcapsules were dropped in the CF-KRH containing 100 mM BaCl_2_ as a crosslinking solution. The voltage and extrusion flow rate were considered 8 kV and 0.08 ml/min, respectively. Continuously, the formed microcapsules were washed by the CF-KRH buffer twice to remove thr unbounded barium ions. Then, both prepared Alginate-Gelatin (Alg/Gel) and Alginate-Gelatin/Nano-Hydroxyapatite (Alg/Gel/nHA) microcapsules were transferred to the distinct flasks containing the culture medium. The flasks were then incubated under the atmosphere of 5% CO_2_ at 37 °C and monitored for 28 days for more cell experiments and morphology evaluation by the microscope (Olympus IX71). The culture medium was refreshed every 3 days. The surface morphology of the microcapsule samples was also observed by scanning the electron microscopy (SEM, Tescan MW2300).

### Proliferation and differentiation of hDPSCs

#### Metabolic activity and live/dead assays

Human dental pulp stem microbeads were used to determining the metabolic activity in 7, 14, and 21 days. 5 mg/ml MTT solution was added to each well. The plates were incubated under the atmosphere of 5% CO_2_ at 37 °C for 4 h. Then, the DMSO was added to wells and the absorbance was measured by the UV-160 spectrophotometer (BioQuest) at 570 nm. The dental pulp stem cells, which were cultured on T-flasks coated by the polystyrene surface (PS) were considered as a control group. The absorbance values were normalized by considering the number of the microcapsules per sample and the experiments were carried out in the triplicates and the data were reported as the mean ± SD.

To live/dead assay, after rinsing microcapsulated cells by PBS, these microcapsules were incubated in the saline containing calcein-AM and propidium iodide (PI) at 37 °C, as described elsewhere [80]. The microcapsules were then washed by PBS three times again after incubating in 5% carbon dioxide for 45 min. Finally, the microcapsules containing hDPSCs were observed under the fluorescence microscope (Olympus, IX71) at the wavelengths of 488 nm (green, living cells) and 543 nm (red, dead cells).

#### Gene expression via qRT- PCR

The osteogenic gene expression was evaluated after 21 and 28 days. After washing microcapsules by the PBS, samples were fractured gently. Then, Trizol reagent was used for the extraction of total RNA according to the manufacture instruction. The gel electrophoresis and Nanodrop (Thermo Scientific, Waltham, MA, USA) were applied for determining the yield and value of the extracted RNA. 1 μl of the total synthesized RNA was used for the cDNA synthesis by CDNA synthesis kit (YektaTajhizAzma, Iran). Synthesized cDNA, syber green master mix, and designed primers were mixed based on the manufacture instruction. Primer sequences, melting temperature, and amplicon size of the designed primers were offered in Table 1. Cells were cultured on the polystyrene culture surface of the T75 flasks for the control group. All the experiments were repeated three times.

**Table 1:** Sequences, melting temperature, and amplicon size of primers used for RT-PCR

Target	Sense and antisense sequences 5′ to 3’	tA (°C)	bp
BMP-2	F: GAGAAGGAGAGGCAAAGAAAG	61/59	183
	R:GAAGCAGCAACGCTAGAGAC		
Osteocalcin	F: ATTGTGGCTCACCCTCCATCA	60	119
	R: AGGGCTATTTGGGCGTCATC		
Osteonectin	F:GCAGAGGAAACCCAAGAGGAG	60	208
	R: TGGCAAAGAAGTGGCAGCAG		
RUNX-2	F:ACCTTGACCATAACCGTCTTC	67/57	145
	R: GGCGGTCAGAGAACAAACTA		
DSPP	F:CTGGTGCATGAAGGTCATAGAG	57	90
	R: CAATTTGCGGATCTCAGAGG		
GAPDH	F: GAAGGTGAAGGTCGGAGTC	65	100
	R: GAAGATGGTGATGGGATTTC		

#### Alkaline phosphatase activity assay

Alkaline phosphatase [71] tests were carried out for the hDPScs microcapsulation after 21 days. For alkaline phosphatase staining, the BCIP/NBT solution was prepared and added over non-medium microcapsules before incubating in a dark situation. Then microcapsules were kept in the formalin solution (10%) for one minute. Finally, the microcapsules were washed by the PBS buffer and photographed. ALP activity was quantified after 21 days microcapsulation, according to the method described elsewhere (44). Briefly, microcapsules were rinsed with the PBS and centrifuged at 1200 rpm for 20 min. Then, the supernatant was collected and the ALP activity was detected by using the ALP assay Kit (DIALAB, Austria) according to the manufacturer protocol. ALP activity was measured by evaluating the absorbance at 405 nm by the spectrophotometer and normalized with total protein content. The experiments were carried out in the triplicate and hDPSCs cultured on the conventional polystyrene culture surface of the T75 flasks was used as the control group.

#### Alizarin red S staining

Calcium deposition of the hDPSCs in the modular structure was assessed by the alizarin red S staining after 21 days. Shortly, the microcapsules were removed from the flask and washed three times by the HEPES buffer and fixed by 3.6% (v/v) formaldehyde solution for 15 min at 25 °C. 2% alizarin red liquid (pH: 4.1–4.3) was then poured over the samples in a dark place and maintained for 15 min. Finally, the stained microcapsules were washed by the deionized water several times and examined under the microscope. ImageJ software 1.52n version was used to analyzing quantitatively the intensity of the stained microcapsules.

#### Statistical analysis

The data were determined as the mean ± standard deviation and comprised by using One-way ANOVA and Tukey Test analysis with Prism software (version 8.0, GraphPad, San Diego, CA, USA). *P*-value< 0.05 was considered statistically significant.

## Data Availability

All data generated and/or analyzed during this study are included in this published article. The datasets used and/or analyzed during the current study are available from the corresponding author on reasonable request.

## Abbreviations

Alg/Gel: Alginate/Gelatin
Alg/Gel/nHA: Alginate/Gelatin/nano-Hydroxyapatite
ALP: Alkaline phosphatase
BMSCs: Bone Marrow Mesenchymal Stem Cells
BTE: Bone Tissue Engineering
hDPSCs: Human Dental Pulp Stem Cells
nHA: Nano-hydroxyapatite
SEM: Scanning electron microscopy
